# Comparison of Gait Characteristics for Horses Without Shoes, with Steel Shoes, and with Aluminum Shoes

**DOI:** 10.3390/ani15162376

**Published:** 2025-08-13

**Authors:** Katherine Gottleib, Lauren Trager-Burns, Amy Santonastaso, Sophie Bogers, Stephen Werre, Travis Burns, Christopher Byron

**Affiliations:** 1 Department of Large Animal Clinical Sciences, Virginia-Maryland College of Veterinary Medicine, Virginia Tech, Blacksburg, VA 24061, USA; kgottleib@vt.edu (K.G.); santonas@vt.edu (A.S.);; 2 Department of Population Health Sciences, Virginia-Maryland College of Veterinary Medicine, Virginia Tech, Blacksburg, VA 24061, USA

**Keywords:** inertial measuring unit, IMU, gait analysis, limb flight, kinematics, hoof arc, shoeing

## Abstract

Differences in horseshoe materials may have effects on gait that could change perceived esthetic qualities. Objective information regarding effects of shoeing on gait characteristics of horses is scant. The aim of this study was to determine differences in gait characteristics for horses under various experimental shoeing conditions (barefoot, aluminum shoes, steel shoes) on two surfaces (asphalt and soft footing) using body- and hoof-mounted sensors. We hypothesized that shoeing would affect hoof arc height during early (arc height a) and late (arc height b) swing phases but would not affect other gait variables. Twelve healthy, adult, client-owned horses were evaluated at a trot on asphalt and soft footing under the three experimental shoeing conditions. No significant (*p* < 0.05) effects of shoeing were detected for gait symmetry (Q score), mediolateral hoof deviation, stride length, or midstance, breakover, swing, and landing stride phase times. Hoof arc height a was significantly (*p* < 0.001) lower for aluminum versus steel shoes for right and left forelimbs on asphalt and soft footing. Hoof arc height b was significantly higher for aluminum versus steel shoes on soft footing for left (*p* < 0.001) and right (*p* = 0.02) forelimbs. Findings indicate that shoe weights affect early and late swing phase hoof heights differently. Further investigation is warranted to determine whether measured hoof arc height changes affect subjective esthetics of gait.

## Simple Summary

Horses often wear metal shoes to provide protection for the hoof and aid traction during training and competitions. The most common metal shoe materials include steel and aluminum, which is lighter than steel and is thought by some competitors to have desirable effects on gait esthetics. There is only a small amount of information available regarding the effects of shoeing on certain aspects of gait, and additional information would be useful. In this study, hoof- and body-mounted measurement devices were used to determine the effects of various shoeing conditions (barefoot, aluminum shoes, and steel shoes) on several aspects of equine gait while trotting on asphalt and soft footing. No significant effects of shoeing were detected for gait symmetry, inside/outside hoof deviation, stride length, or stride phase times. Hoof height during early swing phase of the stride was significantly lower for aluminum versus steel shoes on both asphalt and soft footing. Hoof arc height during late swing phase of the stride was significantly higher for aluminum versus steel shoes on soft footing only. Further investigation would be useful to determine effects of other shoe types and whether these measured hoof arc height changes influence subjective esthetic qualities of gait.

## 1. Introduction

Many equine athletes wear metal shoes to provide protection against surfaces, support the foot, and aid traction. Steel has traditionally been chosen for its durability, availability, and low cost. However, aluminum can be used to manufacture a lighter shoe, which is thought to have desirable effects on gait esthetics [1]. For example, steel shoes may be removed (barefoot conditions) or changed to a lighter shoe material (e.g., aluminum) during hunter competitions in the United States to achieve more esthetic limb flight for portions of the event when this might be advantageous (such as during flat classes judged on subjective qualities of movement); heavier steel shoes may be left in place to improve traction and support during other portions of the competition (such as classes over fences) [2]. Not enough scientific data exist to support or refute concerns over potential effects of shoeing on horses engaged in these events, including impact on performance and perceptions of welfare [2]. Therefore, there is a perceived need for additional scientific studies investigating the effects of certain horseshoe characteristics on gait.

Shoes have effects on gait characteristics, performance, and injury rates of horses in a variety of athletic disciplines [1,3,4,5,6]. The effects of shoe shape and surface on kinetic and kinematic stride variables have been extensively investigated; however, studies evaluating effects of shoe composition or weight are scant [7]. Shoeing type and weight have an effect of breakover duration during the stride; however, these changes are not observed at gaits typical for show horses [8]. Higher hoof unit weights (achieved with shoes or hoof training weights) increase stride duration [9,10] but do not influence stride length [1,9]. Higher hoof unit weights also have effects on joint angles and maximum hoof height during the swing phase of the stride [1,9,11]. Effects on maximum hoof height may be particularly relevant to horses engaged in subjectively judged events, since swing phase of stride is important to esthetic aspects of movement [12]. Although studies to date provide valuable information regarding effects of shoeing on certain gait characteristics, further information (particularly about hoof flight during the swing phase of the stride) would be useful.

Body-mounted sensors comprising accelerometers and gyroscopes (measuring changes in acceleration and angular velocity, respectively) have been used to objectively analyze gait characteristics in horses [13]. The most commonly used body-mounted inertial sensor systems measure gait symmetry using head and pelvic movement [14]. Such systems are highly useful for objective detection of lameness in horses. However, a key limitation to body-mounted systems is that they do not directly measure gait characteristics of the distal limb, such as hoof height and limb arc during swing phase. Such gait characteristics are typically measured using video optoelectronic systems [1], which have limitations in that the capture devices are stationary and location of data capture is therefore limited. Therefore, use of alternative technologies that allow more flexible data acquisition may be desirable.

Limb-mounted inertial measurement unit systems (IMUs) are convenient tools for gait analysis in horses. Other authors comparing fetlock joint angle patterns determined with IMUs and optical motion capture determined that data have high agreement between those methods [15], indicating IMUs are accurate for evaluation of distal limb gait analysis without the limitations of optical motion capture systems including reduced portability and requirement for dedicated space. Recently, triaxial hoof-mounted inertial sensor systems have been shown to be highly accurate for measurement of distal limb stride characteristics in horses [16,17,18]. These sensors yield information about hoof orientation in all three axes and allow more detailed analysis of movements of the distal limb than other technologies. In addition, these sensor systems allow a high degree of flexibility in data acquisition conditions. As a result, accurate data may be collected under a greater variety of experimental settings and with greater efficiency. These advantages make hoof-mounted IMUs particularly well suited for detailed examination of the effects of shoeing on distal-limb gait characteristics.

There remains a lack of enough objective information regarding the effects of horseshoes on certain aspects of equine gait, particularly swing phase characteristics such as hoof arc height. This information may be relevant for veterinarians, farriers, and professionals considering changes in rules for competitions. The objective of this study was to compare objectively measured gait characteristics in horses for various hoof shoeing conditions (barefoot, aluminum shoes, and steel shoes) using hoof-mounted IMUs. We hypothesized that hoof shoeing conditions would not affect symmetry of head and pelvic movements as detected with body-mounted inertial sensors or mediolateral hoof deviation during swing phase, stride length, and duration of stride phases detected with hoof-mounted IMUs. We also hypothesized that lower foot-shoe weight (barefoot or aluminum shoes) would result in lower early and late swing phase hoof flight height (arc height a and arc height b, respectively) as detected with hoof-mounted IMUs.

## 2. Materials and Methods

### 2.1. Animals

A power analysis was used to determine population size for statistical significance (alpha = 0.05; power = 0.80; assumed difference between maximum and minimum values = 20%; assumed standard deviation = 15%). The calculation yielded a suggested sample size of 11 horses; an additional horse was included to account for higher data variability or unanticipated loss of an enrolled horse from the study. Therefore, 12 healthy, client-owned horses without lameness or hoof and limb abnormalities were recruited for the study. Protocols were approved by the Virginia Tech Institutional Animal Care and Use Committee (protocol 22-127). Horses were to be excluded if they had a history of laminitis or performance-limiting orthopedic disease, were sensitive to hoof testers, had received nonsteroidal anti-inflammatory drugs within the prior 7 days, or had an AAEP Lameness Grade greater or equal to 1/5. No abnormalities were detected during physical examination, hoof tester application, lameness assessment, or radiography; therefore, no horses were excluded. Horses included 9 American Quarter Horses and 3 Thoroughbreds. The mean ± SD weight of horses was 479.9 ± 35.8 kg (range, 431.4 kg to 535.5 kg). The horses were used for several different disciplines (western pleasure, eventing, and pleasure riding) and were all in active training and use.

### 2.2. Procedures

A physical examination was performed prior to initiation of the experiments for all 12 horses. Hoof testers were applied to all four feet at the toe margins, at the lateral bars, across the frog, and across the heels. Hoof balance radiographs (lateromedial and dorsopalmar projections) were taken of the front feet prior to the removal of the horses’ own shoes. Horses were trotted in hand on a straight line over asphalt and evaluated by the same observer (CB) to ensure they did not have lameness.

**Figure 1:**
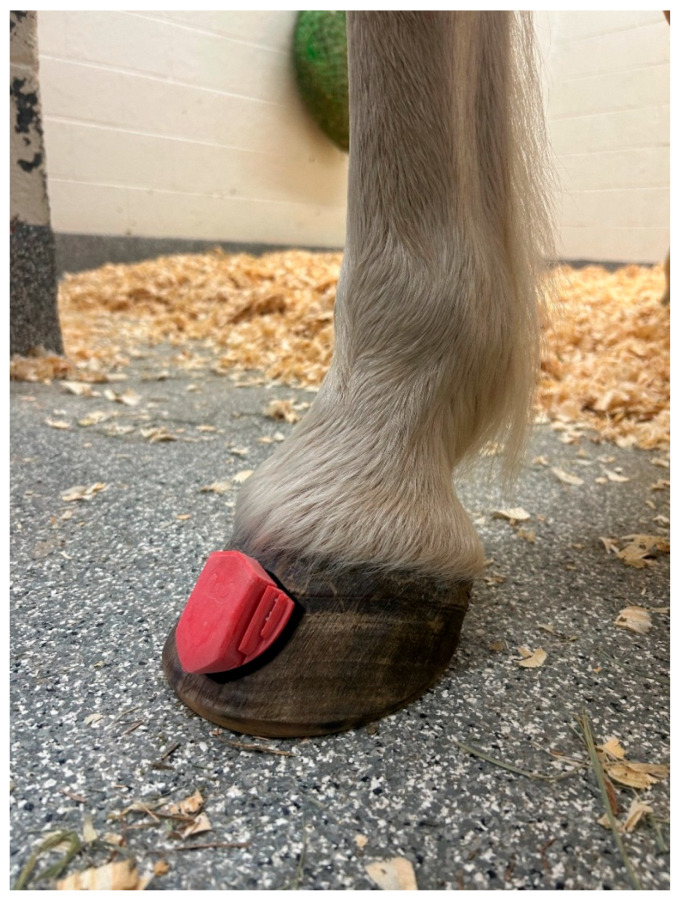
Image of a hoof-mounted inertial measuring unit affixed to the midline on the dorsal aspect of the hoof wall approximately 1 cm distal to the coronary band.

Horses were fitted with a single axis accelerometer fastened to the most dorsal aspect of the poll using a specialized neoprene cap attached to the halter, a second single axis accelerometer fastened to the skin on midline between the tuber sacrale, and a single axis gyroscope attached to the dorsal aspect of the right front pastern in a specialized neoprene strap (The Equinosis Q with Lameness Locator, Equinosis Inc., Columbia, MO, USA). A hoof-mounted inertial measurement unit (IMU; HoofBeats Gait Mapping System, Werkman Black, The Netherlands) was applied to the dorsal aspect of the hoof wall on each forefoot using adhesive and Velcro for repeatability of subsequent placement (Figure 1). Each IMU was placed approximately 1 cm distal to the coronary band on midline of both forelimbs. Data were collected while the horses were trotted in hand on a straight line over asphalt and soft footing (mortar sand over #10 stonedust base). Data were collected for horses before interventions of original shoe removal (i.e., with feet in the condition present at the time of presentation; baseline) and under three experimental conditions in the following order: barefoot, aluminum shoes (Kerckhaert Aluminum Comfort, Kerckhaert Royal Horseshoes, Vogelwaarde, The Netherlands), and steel shoes (Kerckhaert Steel Comfort, Kerckhaert Royal Horseshoes, Vogelwaarde, The Netherlands). For baseline conditions, 11 horses wore steel shoes on forelimbs and 1 horse was barefoot (because shoes had been removed prior to presentation). All trimming and shoeing was performed by the same farrier (TB). Shoes were weighed prior to application; mean ± SD weights were 172.9 ± 19.3 g for aluminum shoes and 372.9 ± 31.6 g for steel shoes. Horses were trotted and data were collected immediately after each shoeing change. Under each condition, the horses were trotted in hand on a straight line over asphalt followed by soft footing; each trial was performed in triplicate for each condition and surface; triplicate trials were performed in immediate succession. Hoof balance radiographs (0° lateromedial and 0° dorsopalmar projections) were obtained to confirm appropriate hoof balance and confirm that no major subjective changes to hoof balance had occurred during shoe placement.

### 2.3. Measures of Outcome

**Figure 2:**
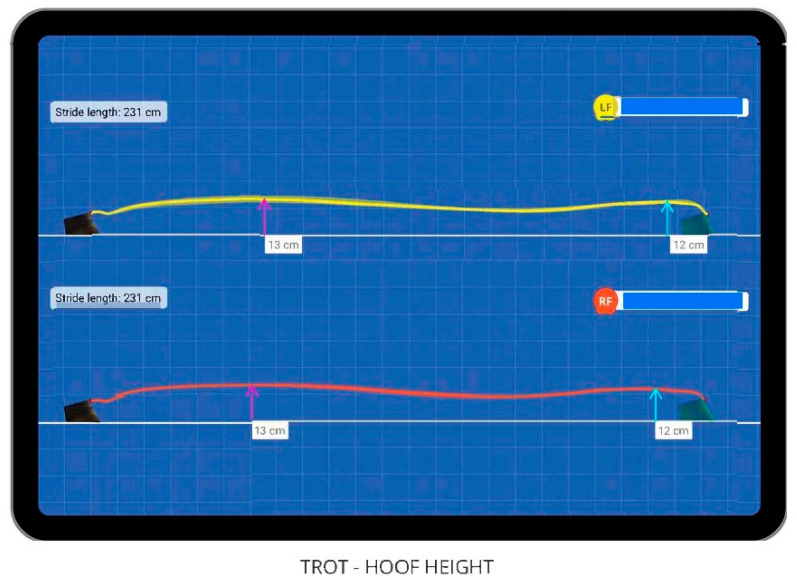
Example of gait map for hoof flight at the trot of left (LF, yellow tracing) and right (RF, red tracing) forelimbs. Two distinct maximum hoof heights are indicated as the maximum arc height during early swing phase (arc height a; fuschia arrows) and maximum arc height during late swing phase (arc height b; cyan arrows) immediately prior to landing.

Data were collected using the hoof-mounted inertial sensors including hoof arc height at two points of limb flight (arc height a [early swing phase] and arc height b [late swing phase]; Figure 2), mediolateral deviation of the hoof flight, stride length, and the time of each phase of stride (landing, midstance, breakover, and swing phases). Two arc height measurements (arc height a and arc height b) identified by the inertial sensor system result from the biphasic path of hoof flight during the swing phase of the stride. In early stance phase, the hoof attains an initial maximum height (arc height a) followed by a period of decreasing height. A second peak in arc height (arc height b) occurs in late swing phase before the initiation of stance phase, when the hoof contacts the ground. Hoof arc heights a and b were direct values from the data output of the inertial sensor system; hoof arc height gait maps were generated as a visual representation and were not used for measurements. Data collected using body-mounted sensors (The Equinosis Q with Lameness Locator) included Q score as a measure of forelimb asymmetry. Q score was calculated based on the vector sum (square root of [HD_max_^2^ + HD_min_^2^], where HD_max_ is the difference in head maximum heights between right and left portions of the stride, and HD_min_ is the difference in head minimum heights between right and left portions of the stride). Q score represents the vector sum for left (HD_min_ negative values) or right (HD_min_ positive values) forelimbs [19].

### 2.4. Statistical Analysis

Measurements from the 3 trials (technical replicates) were averaged using a median. Distribution properties of the data were assessed using normal probability plots. Normally distributed outcomes (stride length and the time of each phase [landing, midstance, breakover, and swing] of stride) were summarized as mean (standard deviation). Non-normally distributed data (hoof arc height at two points of limb flight [arc height a and arc height b], Q score, and mediolateral hoof arc deviation) were summarized as least squares means (standard error). Effects of shoeing (baseline vs. barefoot vs. aluminum vs. steel) and footing (A [asphalt] vs. SF [soft footing]) on outcomes were assessed using mixed-model ANOVA for normally distributed data or linear generalized estimating equations for non-normally distributed data. For all models, the fixed effects were specified as shoeing, footing, and the interaction between shoeing and footing. For mixed-model ANOVA, horse identification was specified as the random effect. For linear generalized estimating equations, horse identification was specified as the subject of repetition with a compound symmetry matrix. Where appropriate, *p*-values were adjusted for multiple comparisons using Tukey’s procedure. Statistical significance was set to *p* < 0.05. All analyses were performed using SAS version 9.4 (Cary, NC, USA).

## 3. Results

### 3.1. Hoof Arc Height

The least squares means and standard error arc height a and arc height b for left and right forelimbs under all conditions (baseline, barefoot, aluminum shoes, and steel shoes) and surfaces (asphalt and soft footing) are reported in Table 1.

#### 3.1.1. Arc Height a

Overall comparisons adjusted for data on all surfaces (asphalt and soft footing) indicated arc height a for left and right forelimbs was significantly (*p* < 0.001) lower for aluminum shoes versus steel shoes.

Left forelimb arc height a on asphalt was significantly lower for aluminum shoes versus steel shoes (*p* < 0.001). Left forelimb arc height a on soft footing was significantly lower for barefoot conditions versus aluminum shoes (*p* = 0.045), barefoot conditions versus steel shoes (*p* < 0.001), barefoot versus baseline conditions (*p* = 0.02), and aluminum versus steel shoes (*p* < 0.001).

Right forelimb arc height a on asphalt was significantly lower for aluminum shoes versus steel shoes (*p* < 0.001) and aluminum shoes versus baseline conditions (*p* = 0.03). Right forelimb arc height a on soft footing was significantly lower for aluminum versus steel shoes (*p* < 0.001) and barefoot conditions versus steel shoes (*p* = 0.03).

#### 3.1.2. Arc Height b

Overall comparisons adjusted for data on all surfaces (asphalt and soft footing) indicated left forelimb arc height b was significantly (*p* = 0.005) greater for aluminum versus steel shoes. Right forelimb arc height b for all surfaces was significantly (*p* = 0.007) greater for barefoot conditions versus baseline conditions.

No significant differences were found for comparisons of left forelimb arc height b on asphalt. Left forelimb arc height b on soft footing was significantly higher for aluminum versus steel shoes (*p* < 0.001).

Right forelimb arc height b on asphalt was significantly higher for barefoot conditions versus aluminum shoes (*p* < 0.001), barefoot versus baseline conditions (*p* = 0.003), barefoot conditions versus steel shoes (*p* < 0.001), and baseline conditions versus steel shoes (*p* = 0.04). Right forelimb arc height b on soft footing was significantly higher for aluminum versus steel shoes (*p* = 0.02) and barefoot conditions versus steel shoes (*p* = 0.048).

### 3.2. Stride Length

The mean and standard deviation stride length under all conditions (baseline, barefoot, aluminum shoes, and steel shoes) and surfaces (asphalt and soft footing) are reported in Table 2. No significant differences were found for comparisons of shoeing conditions on each surface.

### 3.3. Q Score

The least squares means and standard error Q score for left and right forelimbs under all conditions (baseline, barefoot, aluminum shoes, and steel shoes) and surfaces (asphalt and soft footing) are reported in Table 3. No significant differences were found for comparisons of shoeing conditions on each surface.

### 3.4. Mediolateral Deviation of the Hoof and Time of Each Stride Phase

The least squares means and standard error mediolateral hoof deviation in swing phase of the stride for left and right forelimbs under all conditions (baseline, barefoot, aluminum shoes, and steel shoes) and surfaces (asphalt and soft footing) are reported in Table 4. No significant differences were found for comparisons of mediolateral hoof deviation.

The mean and standard deviation duration of phases of the stride (landing, midstance, breakover, and swing) for left and right forelimbs under all conditions (baseline, barefoot, aluminum shoes, and steel shoes) and surfaces (asphalt and soft footing) are reported in Table 5. No significant differences were found for comparisons of stride phase time among shoeing conditions for each surface.

## 4. Discussion

The purpose of this study was to quantify effects of three different shoeing conditions (barefoot, aluminum shoes, and steel shoes) on hoof flight and stride characteristics of horses using hoof-mounted inertial measurement units (IMUs). We hypothesized that hoof shoeing conditions would not affect symmetry of head and pelvic movements as detected with body-mounted inertial sensors (Q score), or mediolateral hoof deviation during swing phase, stride length, and duration of stride phases as detected with hoof-mounted IMUs. These hypotheses were supported by findings of the study; results indicated shoeing conditions did not significantly change gait symmetry or those other measured gait characteristics.

We also hypothesized that lower shoeing weights (barefoot or aluminum shoes) would result in lower hoof height during early swing phase (hoof arc height a) compared with steel shoes. This hypothesis was fully supported for comparisons of aluminum and steel shoe data, but only partially supported for comparisons of barefoot conditions data. Results showed that arc height a was significantly lower for aluminum shoes versus steel shoes for both limbs on all surfaces. These results are similar to those of Huguet et al. [1], who found that maximum hoof height is lower for horses wearing aluminum shoes versus steel shoes. Horses in that study were only trotted on asphalt. A novel finding of our study was that the relative early swing phase hoof arc height difference between aluminum and steel shoes is also found for horses trotting on soft footing, which more closely mimics conditions typical for horses during most types of showing. It is not surprising that differences in weight between aluminum and steel shoes would contribute to differences in limb flight, since the most influential variable on motion of limbs during swing phase of the stride is hoof unit weight (to which shoes contribute substantially) [20,21]. Our results also indicated arc height a was significantly lower for barefoot conditions versus horses with aluminum (left forelimbs) and steel (right and left forelimbs) shoes on soft footing. It is interesting to note arc height a for barefoot conditions was higher than aluminum shoe values and lower than steel shoe values on asphalt, although these differences were not significant. Differences in results between asphalt and soft footing may have been attributable to the effects of surface type on gait characteristics [22]. Neural feedback from hoof mechanoreceptors [23] could also contribute to differences in gait between hard and soft surfaces. Although feedback from the hoof does not seem to affect stance phase variables [24], effects on swing phase characteristics are unknown. Findings of right–left asymmetry could be attributable to handlers always leading horses from the left side or to inherent laterality or asymmetry of the horses [24,25].

The hypothesis that lower shoeing weights (barefoot or aluminum shoes) would result in lower hoof flight height during late swing phase (hoof arc height b) was not supported. Arc height b was significantly higher for horses wearing aluminum shoes versus steel shoes on soft footing. This novel finding was likely attributable to differences in gait mechanics between early and late swing phases of the stride. Limb movement during early swing phase is initiated by elbow flexors on the cranial side, resulting in inertial forces on the distal limb [26]. During late swing phase, caudal muscle activity leads to contraction and lowering of the limb toward the ground [20,26]. This sudden shift in forces on the lower limb changes the shape of hoof flight at the end of swing phase. Different shoe weights likely result in different shape, timing, and height of hoof flight.

These findings may have implications for both the clinical approach to shoeing horses and the regulations surrounding equine sportsmanship. Because maximum early swing phase hoof height (arc height a) was higher and late swing phase hoof height (arc height b) was lower when horses wore steel shoes versus when they wore aluminum shoes, the difference between these two arc heights is greater for horses wearing steel shoes. It is possible that this small difference in arc heights could be detected and subjectively interpreted differently by an observer (judge), although additional studies would be required to determine the minimum detectable change in arc height that could be visually detected under conditions similar to those in this study. This change to the shape or appearance of the biphasic hoof flight during swing phase of the stride is a novel finding and may have observable effects on esthetic qualities of gait [12]. Research into human observation of gait asymmetry compared with sensor-based system measurement data found a threshold of observation by the human eye to be a difference in symmetry of >25% [27]. The measured differences in least square means of hoof arc height between shoeing conditions of this study range from 0.6 to 2.5 cm, a magnitude of < 20% of mean values; although that difference might be detectable by an observer under certain conditions, it is less than the visual detection threshold of 25% [27]. Other investigators used distal limb IMU measurements of limb flight to identify phenotypes of Italian horses used for dressage or draft work [28]. The researchers in that study compared the perceived limb flight and gait quality of trained equine judges to the data collected from an IMU sensor-based system and concluded that sensor data could accurately predict the evaluation of gait quality by the judges and demonstrated the potential use of the technology as an assessment of performance. Although results of our study do not indicate a change in hoof flight that would be visually detected, this idea merits further consideration in future work.

The magnitude of differences in hoof flight found in our study are similar to those found by other investigators, who detected a 2.5 to 3 cm decrease in maximum swing phase hoof height for horses with lower hoof unit weights [1,11]. Another common finding is that shoe materials do not influence stride length at the trot [1]. Doubling the weight of shoes increases the hoof arc height during swing phase in horses [29]. Similarly, steel shoe weights in our study were slightly more than twice the weight of aluminum shoes. Although weight is only one variable between different types of shoes, it is likely the major cause of limb flight differences because shoe weights, but not configurations, have been shown alter swing phase characteristics [20].

Results of this study indicated that, while hoof arc height was influenced by shoeing, other measures of gait and symmetry of movement were not affected. Measures of head and pelvis asymmetry (Q score) collected using body-mounted sensors were not significantly different among shoeing condition groups. This finding agrees with results of another study in which no statistical difference in total head height and pelvis movements were found before or after farrier intervention [6]. Our results and those of the aforementioned study indicate trimming and shoeing with various materials do not have an immediate effect on gait symmetry of horses. Also, other than hoof arc height, no other measured variables of gait (stride length, mediolateral hoof deviation during swing phase, or time of stride phases) were significantly different among shoeing conditions, similar to findings of other authors [1]. These results suggest that, for experimental conditions in this study, the effects of shoe material and weight are primarily limited to the shape and height of hoof arc height during swing phase of the stride.

Data acquired before farrier intervention (baseline) were used as a measure of initial gait characteristics and comparisons of these data were not included in our hypotheses. Because 11 horses wore steel shoes and 1 was barefoot during baseline data collection (since that is the condition in which they were presented), definitive conclusions regarding comparisons of baseline and other conditions cannot be made.

The hoof-mounted IMU used in this study allowed detailed measurement of several variables of gait at the trot. These IMUs have excellent agreement with optoelectronic methods for measurement of variables associated with landing, breakover, stance, and swing phases of the stride [16]. In fact, optoelectrical capture resolution may not have sufficient resolution to delineate subtle differences among experimental conditions [16,20]. Use of IMUs in our study was convenient and provided data with high resolution that allowed detection of relatively small hoof flight differences among groups.

Limitations of this study include the low sample size (*n* = 12) of horses. Although a power analysis was used to determine the population size, additional comparisons may have had statistically significant differences if a larger population had been used. Also, horses enrolled in this study were involved in training for disciplines of western pleasure and eventing. The practice of removing shoes mid-competition is currently adopted by some regional competitors in the hunter discipline [2]. Unfortunately, the authors did not have access to a large population of horses engaged in that discipline for recruitment in this study. In addition, since horses enrolled in the study typically wore steel shoes because of owner preference, the experimental order (barefoot, aluminum shoes, then steel shoes) allowed horses to be discharged from the study wearing their typical shoe without additional resetting; this order does not represent the order in which shoeing is typically manipulated during hunter competitions and is a potential limitation. Furthermore, collection of data at the trot in our study does not represent all gaits commonly used in equestrian events, such as the canter and working canter. Therefore, it is not appropriate to extrapolate these results for horses working at gaits other than the trot or for horses working in circles and over jumps. In this study, all horses were trotted on asphalt first, followed by soft footing. This lack of randomization could have affected the results if horses became more fatigued throughout the day. Additionally, it is possible the horses were able to adjust to the weight of a new shoeing condition over asphalt, which may have altered their gait over soft footing. Further studies should incorporate randomization to strongly corroborate the data from the current study.

The hoof-mounted IMUs used in this study are lightweight, easy to apply, and data acquisition is straightforward. In future studies, this system could be applied and used in simulated competition settings for horses undergoing changes in shoeing conditions between event types, which is commonplace in some disciplines. Additionally, such data could be compared to subjective judging scores to determine correlations between hoof flight and esthetic preferences of judges. Although other authors have determined that objective measurements may be useful for fitness-to-compete judgements [30,31], few studies have been conducted comparing objective analysis metrics of horse gait to subjective esthetic judging outcomes [28]. Additional studies investigating such comparisons would enable determination of the possible effects of shoeing conditions on show performance and identification of the advantages of shoeing changes during competition. Further studies could also be performed to determine effects of shoeing condition changes on horse welfare, particularly hoof health and related lameness.

## 5. Conclusions

In conclusion, results of this study identified alterations in hoof flight (hoof arc height) among shoeing conditions, particularly in early stance phase and between aluminum and steel shoes. The data shows a significant increase in overall arc height a (early swing phase arc height) under the heavier shoe conditions (steel) compared to lighter shoe conditions (barefoot or aluminum shoes), regardless of the footing type. In addition, horses typically had lower arc height b (late swing phase arc height) when wearing heavier steel shoes on soft footing. Further studies are warranted to investigate effects of other shoeing types on gait characteristics and subjective judging outcomes. Results of this study may be relevant for equine professionals involved in the care and regulation of sport horses. Further research should be performed to confirm these findings for horses performing in specific disciplines, especially hunters.

## Figures and Tables

**Table 1:** Least squares means (SE) for hoof height from the ground at early swing phase maximum (arc height a) and late swing phase maximum (arc height b) for 12 horses before intervention (baseline) and under barefoot, aluminum shoe, and steel shoe conditions. Data are presented for horses trotting on asphalt (A) and soft footing (SF) for left (LF) and right (RF) forelimbs. Data are reported in centimeters. *p*-values are reported for comparisons within each row. Values within each row that have different letters are significantly (*p* < 0.05) different.

Variable	Footing	Baseline	Barefoot	Aluminum	Steel	*p*-Value
LF Arc height a	A	14.00 (0.54) ^ab^	13.38 (0.73) ^ab^	12.88 (0.40) ^b^	14.42 (0.46) ^a^	<0.001
	SF	15.00 (0.46) ^ab^	13.38 (0.69) ^c^	14.75 (0.52) ^b^	15.92 (0.51) ^a^	<0.001
RF Arc height a	A	14.29 (0.74) ^a^	13.42 (0.73) ^ab^	12.88 (0.49) ^b^	14.42 (0.61) ^a^	<0.001
	SF	14.71 (0.45) ^ab^	13.92 (0.75) ^b^	14.54 (0.50) ^b^	15.75 (0.49) ^a^	<0.001
LF Arc height b	A	12.08 (0.30) ^a^	12.38 (0.35) ^a^	12.04 (0.27) ^a^	11.67 (0.21) ^a^	0.058
	SF	13.83 (0.44) ^ab^	14.13 (0.44) ^ab^	14.42 (0.48) ^b^	13.71 (0.42) ^a^	<0.001
RF Arc height b	A	12.25 (0.27) ^b^	12.88 (0.39) ^a^	11.92 (0.25) ^bc^	11.67 (0.22) ^c^	<0.001
	SF	13.75 (0.48) ^ab^	14.08 (0.43) ^b^	14.25 (0.46) ^b^	13.63 (0.40) ^a^	0.031

**Table 2:** Mean (SD) stride length in 12 horses before intervention (baseline) and under barefoot, aluminum shoe, and steel shoe conditions. Data are presented for horses trotting on asphalt (A) and soft footing (SF). Values are reported in centimeters. *p*-values are reported for comparisons within each row.

Footing	Baseline	Barefoot	Aluminum	Steel	*p*-Value
A	208.67 (20.40)	206.58 (28.22)	208.13 (20.71)	204.25 (21.89)	0.75
SF	212.63 (14.68)	215.58 (20.93)	217.29 (18.04)	214.42 (14.58)	0.75

**Table 3:** Least squares means (SE) for forelimb Q score in 12 horses before intervention (baseline) and under barefoot, aluminum shoe, and steel shoe conditions. Data are presented for horses trotting on asphalt (A) and soft footing (SF). *p*-values are reported for comparisons within each row.

Footing	Baseline	Barefoot	Aluminum	Steel	*p*-Value
A	10.10 (2.21)	13.73 (2.26)	11.63 (2.23)	11.55 (2.34)	0.26
SF	11.10 (1.49)	9.72 (0.95)	12.18 (2.68)	9.42 (2.20)	0.63

**Table 4:** Least squares means (SE) for mediolateral hoof deviation during the swing phase of the stride of left and right forelimbs in 12 horses before intervention (baseline) and under barefoot, aluminum shoe, and steel shoe conditions. Data are presented for horses trotting on asphalt (A) and soft footing (SF). Values are reported in centimeters. *p*-values are reported for comparisons within each row.

Variable	Footing	Baseline	Barefoot	Aluminum	Steel	*p*-Value
LF Mediolateral hoof deviation	A	4.96 (0.89)	6.08 (0.89)	4.88 (0.61)	4.58 (0.63)	0.06
	SF	4.04 (0.55)	4.00 (0.61)	3.88 (0.52)	3.83 (0.51)	0.91
RF Mediolateral hoof deviation	A	4.71 (1.04)	6.29 (1.08)	5.17 (0.73)	4.67 (0.86)	0.29
	SF	3.38 (0.53)	3.92 (0.78)	3.88 (0.76)	3.54 (0.59)	0.53

**Table 5:** Mean (SD) for durations of each phase of the stride (landing, midstance, breakover, and swing) for left and right forelimbs in 12 horses before intervention (baseline) and under barefoot, aluminum shoe, and steel shoe conditions. Data are presented for left (LF) and right (RF) forelimbs in horses trotting on asphalt (A) and soft footing (SF). Values are reported in milliseconds. *p*-values are reported for comparisons within each row.

Stride Phase	Limb	Footing	Baseline	Barefoot	Aluminum	Steel	*p*-Value
Landing	LF	A	13.7 (3.8)	13.4 (5.1)	12.3 (1.7)	15.2 (2.1)	0.96
		SF	71.8 (15.5)	66.0 (15.0)	76.2 (20.2)	68.7 (18.3)	0.19
	RF	A	15.0 (3.8)	12.9 (4.3)	14.1 (2.7)	15.7 (2.4)	0.95
		SF	67.7 (12.3)	68.4 (20.0)	70.2 (19.8)	66.8 (19.3)	0.54
Midstance	LF	A	245.6 (32.5)	258.9 (39.6)	256.5 (34.8)	262.2 (35.7)	0.11
		SF	223.7 (26.0)	229.3 (26.9)	228.8 (28.3)	234.5 (25.2)	0.51
	RF	A	242.2 (32.7)	254.6 (38.5)	252.8 (36.9)	258.3 (35.2)	0.16
		SF	224.7 (28.8)	226.5 (28.3)	230.9 (29.5)	235.5 (27.8)	0.47
Breakover	LF	A	71.9 (13.3)	73.8 (17.8)	73.6 (12.9)	77.1 (11.9)	0.45
		SF	64.9 (14.6)	61.9 (11.9)	59.7 (12.9)	64.1 (13.7)	0.39
	RF	A	70.0 (12.0)	73.3 (16.8)	72.1 (9.5)	76.6 (12.2)	0.22
		SF	62.3 (11.8)	59.0 (12.3)	58.0 (12.3)	63.0 (11.6)	0.31
Swing	LF	A	369.3 (24.8)	361.6 (24.8)	372.0 (24.8)	372.9 (26.0)	0.06
		SF	375.1 (24.2)	375.9 (24.7)	375.2 (25.8)	379.3 (26.1)	0.75
	RF	A	373.3 (26.2)	366.0 (23.6)	375.6 (25.7)	376.7 (27.4)	0.10
		SF	384.5 (19.4)	382.4 (27.1)	382.3 (29.6)	385.8 (26.3)	0.84

## Data Availability

The data presented in this study are available on request from the corresponding author.
